# Secondary sclerosing cholangitis following extracorporeal membrane oxygenation for acute respiratory distress in polytrauma

**DOI:** 10.1002/ccr3.1660

**Published:** 2018-08-02

**Authors:** Ruth Tunney, Jennifer Scott, Velauthan Rudralingam, Sue Liong, Varinder S. Athwal

**Affiliations:** ^1^ Wythenshawe Hospital Manchester University Hospitals NHS Foundation Trust Manchester UK; ^2^ Divsion of Diabetes, Endocrine and Gastroenterology Faculty of Biology, Medicine & Health University of Manchester Manchester UK

**Keywords:** critically ill, extracorporeal membrane oxygenation, extracorporeal membrane oxygenation, sclerosing cholangitis, SSC‐CIP

## Abstract

Secondary sclerosing cholangitis is a recently identified phenomenon affecting the biliary tree. A subtype has been described in critically ill patients (SSC‐CIP). However, underlying mechanisms are unknown, and few cases have been reported following extracorporeal membrane oxygenation (ECMO). We present a 19‐year‐old male with SSC‐CIP after ECMO following major trauma.

## 1 INTRODUCTION

Secondary sclerosing cholangitis involves progressive destruction of the biliary tree with an identifiable cause, a factor that distinguishes it from idiopathic primary sclerosing cholangitis. When the identified trigger is a period of critical illness, the condition is termed secondary sclerosing cholangitis in the critically ill patient (SSC‐CIP). This is characterized by persistent cholestasis, biliary cast formation, and a structuring, fibrosing cholangiopathy. This condition can result in biliary cirrhosis with liver transplantation as the only definitive treatment.

The earliest biochemical sign of SSC‐CIP is persistent cholestasis. Conversely, cholestasis is a common finding in the critically ill, with causes other than SSC, including drugs, sepsis, and parenteral nutrition. The pathogenesis of SSC‐CIP is still incompletely understood, and cases in a broad range of critically ill patients have been reported.^1^, ^2^, ^3^

Extracorporeal membrane oxygenation (ECMO) provides a means of oxygenating blood in the setting of refractory but potentially reversible respiratory failure when mechanical ventilation is insufficient. There are a number of conditions for which this method has been successfully used in adults. A handful of cases of SSC‐CIP in patients who have received ECMO have been reported, predominantly after ECMO for H1N1 influenza. The combined rarity of SSC‐CIP and the infrequency of ECMO as a treatment in critical illness mean that the case we describe here adds a valuable contribution to the collective experience of this condition.

## 2 CASE REPORT

A 19‐year‐old male of Caucasian origin was admitted to our center as a polytrauma after a road traffic accident. He was previously fit and well, a nonsmoker with an alcohol intake of approximately 10 units per month. The accident, in which his motorcycle collided with an oncoming vehicle, caused him to sustain multiple significant injuries including unstable pelvic fractures and femoral fractures. He had bilateral pneumothoraces, extensive pulmonary contusion, and a splenic hemorrhage. He presented in extremis with signs of hypovolemic shock. He was intubated and resuscitated using local major hemorrhage protocols to achieve a blood pressure of 159/93, receiving ten units of packed red cells and four units of fresh frozen plasma in the emergency department.

He underwent an emergency laparotomy and splenectomy and was subsequently transferred to the intensive care unit, where he became increasingly hypoxic with features of adult respiratory distress syndrome (ARDS). This culminated in him receiving veno‐venous extracorporeal membrane oxygenation (ECMO) from day 15 of his admission for 21 days. He returned to theater on day 21 for a massive haemothorax which required an emergency thoracotomy. After being decannulated from the ECMO circuit, he was stepped down to the general intensive care unit on day 36 and was transferred to the ward on day 55 before being discharged after a 4‐month admission including a prolonged rehabilitation and recovery period.

**Figure 1:**
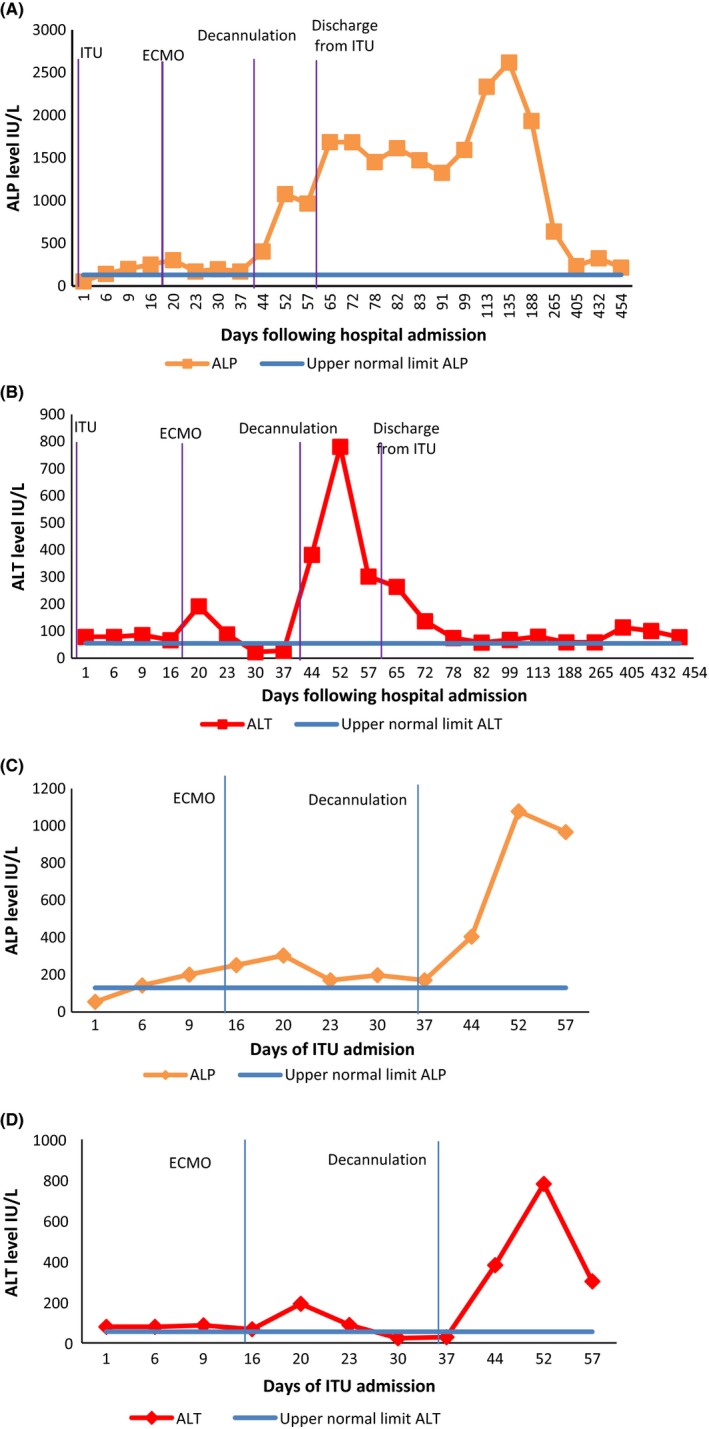
Trends of (A) ALP and (B) ALT since presentation, including outpatient follow‐up. More detailed trend of (C) ALP and (D) ALT during ITU stay only

After presentation and commencement of ECMO, there was a relatively modest change in liver function tests. Alkaline phosphatase (ALP) increased from 55 to 143 IU/L between day 1 and day 6 of hospital admission and no persistent alanine transaminase (ALT) rise until after decannulation. Proceeding decannulation, there was a sequential increase in ALP peaking at 2335 IU/L on day 113. ALT rose to a lesser extent, peaking at 781 IU/L on day 52. The bilirubin did not rise above 57 μmol/L. The pattern of liver function tests is summarized in Figure 1. Autoantibody screen, immunoglobulins, and viral hepatitis serology were negative.

**Figure 2:**
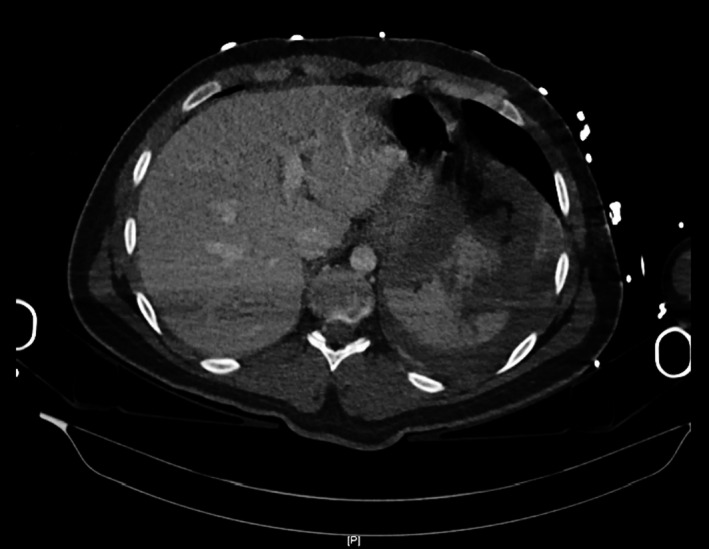
Computed tomography (CT) abdomen done at presentation. No liver abnormality seen on this scan as exemplified by this image

**Figure 3:**
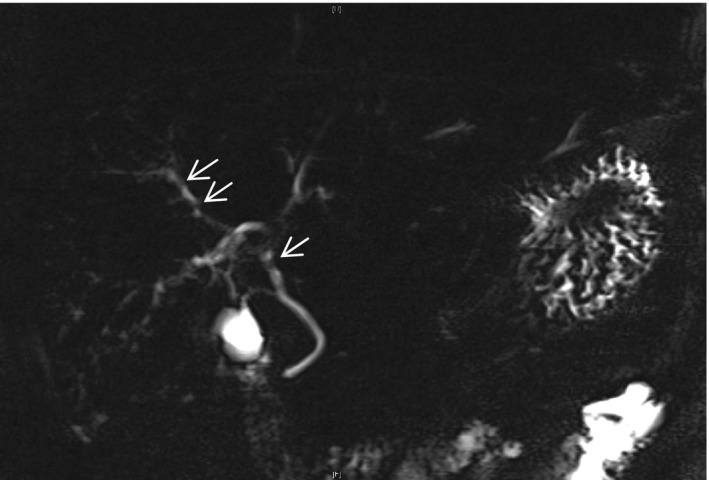
Follow‐up MRCP after outpatient review. Arrow highlights beading and multistenotic pattern of disease

Serial ultrasound, computerized tomography (CT), and magnetic resonance imaging excluded biliary stones and sludge. The liver on CT at day 1 showed normal liver and biliary structure (Figure 2), and it was not until 10 months after the admission, the repeat magnetic resonance cholangiopancreatography (MRCP) demonstrated a multistenotic pattern of disease within the intrahepatic ducts (Figure 3).

In the absence of significant casts within the biliary tree and no evidence to suggest biliary sepsis, endoscopic retrograde cholangiopancreatography (ERCP) was felt not to be helpful. A conservative management approach was taken, and the patient was instigated on ursodeoxycholic acid to help improve cholestasis. With bilirubin improving, the patient was closely monitored as an outpatient upon discharge for progressive liver disease and dysfunction. Repeat imaging and noninvasive fibrosis assessments were undertaken. Despite having evidence of SSC, the synthetic liver function has remained excellent, cholestasis markers have improved, and there have been no episodes of cholangitis or biliary sepsis for over 1 year.

## 3 DISCUSSION

A clinical presentation of secondary sclerosing cholangitis in critically ill patients (SSC‐CIP) has been identified recently.^1^, ^2^ This is characterized by persistent and rapidly progressive cholestasis in the context of critical or life‐threatening illness, resulting in chronic liver injury despite resolution of the causative insult. Clinical findings additional to progressive cholestatic liver function tests are biliary casts seen on ERCP, similar to recipients of orthotopic liver transplant.^4^

The pathogenesis of SSC‐CIP remains unclear, but prevailing opinion is that the insult is hypoperfusion of the biliary vasculature in the first instance. The intrahepatic cholangiocyte epithelium receives its vascular supply from the peribiliary vascular plexus, arising from the hepatic arteries. While the hepatic parenchyma receives a dual blood supply from the portal circulation, a dedicated vascular supply makes the biliary epithelium far more susceptible to variations in arterial perfusion and therefore ischemic injury.^5^ Hence, splanchnic hypoperfusion, irrespective of the etiology, may cause transient visceral ischemia. Given their singular blood supply, biliary cholangiocytes are particularly susceptible to injury secondary to this. The ensuing cholangiocyte dysfunction and necrosis sets in motion a chain of pathophysiologic events, driven by disruption of bile flow and altered bile composition, which culminates in collagen‐rich biliary cast formation.^6^, ^7^ Gelbmann et al^4^ have postulated that ischemic injury of the bile duct is one of the earliest precipitating events of bile duct cast formation.

Several associations have been identified with the development of SSC‐CIP. The common factor is severe, critical illness requiring intensive care treatment. Case studies in the literature have reported SSC‐CIP following severe burns, polytrauma, major surgery, sepsis, and acute respiratory distress syndrome (ARDS), among others.^1^, ^8^, ^9^

The use of ECMO and development of SSC‐CIP is less well reported. Previous case reports of SSC‐CIP have been in patients treated with ECMO following influenza infection.^3^ Here, we describe a case of SSC developing after VV‐ECMO following polytrauma. Critically progressive cholestatic abnormalities were recorded shortly after commencement of ECMO (Figure 1). Mechanical ventilation, hypovolemic shock, and polytrauma correlate less well with the biochemical changes, in contrast to previous studies where they were thought to be causative.^4^ Although this does not conclusively attribute ECMO as the driver of injury, in conjunction with the critical illness state, there does appear to be a causal link.

In many cases of SSC‐CIP, the initial change in liver function tests is the first sign of the development of sclerosing cholangitis, and the biochemical time course of SSC‐CIP is beginning to be established. Cholestatic derangement of liver function is seen, with an initial rise in gamma‐glutamyl transpeptidase, followed by a markedly elevated alkaline phosphatase and a more moderate rise in bilirubin. This bilirubin rise has been observed to fall again without intervention after 2‐6 months. Some cases report aminotransferases show no or minimal elevation secondary to cholestasis,^1^, ^4^ whereas others report a significant rise from early in the presentation.^2^, ^3^

In the early stages, filling defects and biliary casts are present at ERCP and histology demonstrates inflammatory infiltrates. In the first weeks, bile duct structuring is occasionally observed, with progressive segmental biliary duct dilatation that progresses rapidly. As shown in our report, index MRCP demonstrated very limited changes to the intrahepatic biliary tree architecture, despite significant cholestatic biochemistry. The classic beaded appearance seen at endoscopic retrograde cholangiopancreatography (ERCP) favors the intrahepatic ducts and spares the common bile duct and cystic duct. These are seen after 12 months, much like the changes we describe (Figure 1) on subsequent MRCP imaging.^4^, ^10^

Biliary cast formation is a hallmark of SSC‐CIP which is rarely seen in other sclerosing cholangiopathies, and thus, the presence of casts within the intrahepatic ducts may prove a useful differentiating factor when considering other causes of sclerosing cholangitis.^10^ Early cholangiogram to assess the burden of casts within the biliary tree is our practice in suspected cases. If, as in this reported case, only minimal amounts of casts are seen on MR liver with cholangiogram and there is no suggestion of biliary sepsis, we do not routinely proceed to ERCP.

The management of SSC‐IP is limited to reducing cast burden and promoting biliary flow; cast extraction improves liver function tests, but does not impact on the progressive nature of the disease.^4^ Antibiotics can manage infection, and ursodeoxycholic acid is sometimes used with limited efficacy.^11^ None of these measures are certain to alter the progressive course of the disease, and liver transplantation may need to be considered. Post‐transplant survival is comparable to other favorable indications, such as alcohol‐induced cirrhosis.^9^

In an era where extracorporeal oxygenation is being used with increasing frequency, this is a presentation that may become more frequent in centers offering this expertise. Ventricular assist devices, cardiopulmonary transplantation, and acute cardiopulmonary failure have increased experience and use of such technology. In our center, offering all such interventions, presentations as described herein have become increasingly common. There is limited literature regarding optimal management in these cases and as such it is important to report cases.

## 4 CONCLUSION

Here, we have described a case of SSC‐CIP in which ECMO was used for a prolonged period of time. The outcome in this case was excellent, with the patient making a near complete recovery. The contribution of the ECMO itself to the SSC remains undetermined. The biochemical changes occurred after the instigation of ECMO and not mechanical ventilation, supporting ECMO as causative factor. Presently, there are no comparative studies to provide a causal link. Nonetheless, this report adds to the available evidence. Further research is needed to prove this association and to understand the pathology.

## AUTHORSHIP

RT and JS: wrote the manuscript. VR and SL: interpreted radiographic images and production of manuscript figures. VSA: treating physician and conceived and edited the manuscript.

## CONFLICT OF INTEREST

Nothing to report.
